# The burden of schistosomiasis among school-aged children in Ogoja, Nigeria: current level of infection years after mass drug administration with Praziquantel

**DOI:** 10.4314/ahs.v24i4.9

**Published:** 2024-12

**Authors:** Edema E Imalele, Ekanem I Braide, Ubleni E Emanghe, Chinyere Osondu-Anyanwu

**Affiliations:** 1 Department of Zoology and Environmental Biology, Faculty of Biological Sciences, University of Calabar, Calabar, Cross River State, Nigeria; 2 Department of Medical Microbiology/Parasitology, University of Calabar Teaching Hospital, Calabar, Cross River State, Nigeria; 3 Department of Science Laboratory Technology, Faculty of Biological Sciences, University of Calabar, Calabar, Cross River State, Nigeria

**Keywords:** School attendance, schistosomiasis, school-age children, urogenital schistosomiasis, intestinal schistosomiasis

## Abstract

**Background:**

Despite various chemotherapy efforts and national-level regulations implemented by the Nigerian government and health sector stakeholders, Schistosoma infections persist as a public health issue.

**Objective:**

This study assessed schistosomiasis prevalence among school-aged children in Ogoja Local Government Area, Cross River State, and identified risk factors for the disease.

**Methods:**

Urine and faecal samples were examined using microscopy involving centrifugation and Kato-Katz techniques respectively.

**Results:**

The overall prevalence of schistosomiasis was 9.7% (49/504). The prevalence of schistosomiasis was 10.8% and 8.7% among females and males, respectively. Schistosoma haematobium infection was higher in the 14-16 year age group (12.7%). Overall mean parasite load for urogenital schistosomiasis was 6.40 eggs/10 mL of urine and 36.00 eggs per gram (EPG) for intestinal schistosomiasis. Infection with schistosomiasis was higher among those who had not heard about schistosomiasis (17.8%) (p=0.000) and who did not know the cause of the infection (12.4%) (p=0.002). Swimming/bathing in open water (OR = 1.199), fetching water from streams/rivers (OR = 1.202), parents/guardians who had no formal education (OR = 2.722) and unemployment (OR = 2.904) were risk factors significantly associated with schistosomiasis (p P<0.05).

**Conclusion:**

Although intensities of infections were generally low, prompt integrated control efforts are still required to lower helminth infection in the study area.

## Introduction

Schistosomiasis is a parasitic Neglected Tropical Disease (NTD), and over 200 million individuals are infected with Schistosoma species, with 85% of cases occurring in Africa^1^. Estimates show that, in sub-Saharan Africa, approximately 192 million individuals are infected with schistosomiasis^2^. Globally, Nigeria records the highest number of schistosomiasis cases^3^ with researchers reporting a varying prevalence of schistosomiasis^4^-^6^. Urogenital schistosomiasis (caused by Schistosoma haematobium) and intestinal schistosomiasis (caused by S. mansoni) have been established to be associated with chronic and incapacitating conditions occurring in locations of extreme poverty, particularly amongst rural poor and underprivileged urban inhabitants characterised by poor sanitation^7^. School-aged children are most severely infected with intestinal parasites and Schistosoma haematobium^8^. Schistosomiasis negatively impacts a child's development, physical fitness, school attendance, and cognitive function^9^,^10^

The World Health Organization approved praziquantel-based preventive chemotherapy as the primary method of schistosomiasis control in 2001. The main element of this plan was the regular provision of antihelmintic medications to at least 75% of school-aged children^11^,^12^. The Nigerian National Schistosomiasis Control Programme was launched in 1988 and is supported by praziquantel donations from Merck KGaA Germany and Johnson & Johnson. Targeted treatment using praziquantel is currently being conducted in multiple states across Nigeria^13^. Additionally, the Neglected Tropical Diseases (NTDsection of the Cross River State Ministry of Health began its first state-wide school-based deworming campaign in 2016 utilizing praziquantel to treat schistosomiasis. Despite the use of chemotherapeutic techniques, investigations in Nigeria have shown a significant continuous incidence of schistosomiasis^14^-^16^. In a study performed in Ogoja Local Government Area, Cross River State, Adie et al.,^17^ reported that 43.3% of females had blood in their urine. They also stated that Schistosoma infection was found mostly in the Northern and Central parts of Cross River state where the population engages in intensive agricultural practices notably paddy rice cultivation.

Despite several chemotherapeutic initiatives implemented at the national level by the Nigerian government and other health sector stakeholders, Schistosoma infections continue to remain a public health concern. Integrated control strategies that incorporate access to sanitation and other complementary primary preventive activities are required to halt transmission and eradicate schistosomiasis locally^18^. There is a shortage of information on the demographics and sanitary conditions of school-age children in most schools across Nigeria, which might be used to assist establish school health programs. This is necessary for long-term schistosomiasis control in schoolchildren^19^ This study hence determined the current level of schistosomiasis among school-aged children in Ogoja Local Government Area, Cross River State, by assessing the prevalence of infections to suggest recommendations for future control efforts. Risk factors for schistosomiasis in the area were also assessed, as this is important in implementing integrated control measures.

## Materials and methods

### Study site

**Figure 1:**
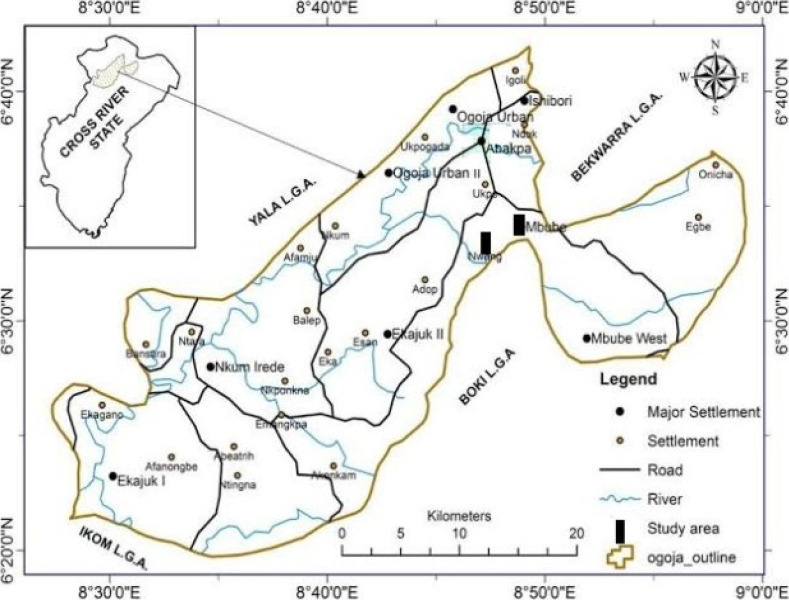
Map of Ogoja Local Government Area showing the different sampling points

The study was conducted in Ogoja Local Government Area (LGA), Cross River State, Nigeria (Figure 1) located on Latitude 6039′17″ North and Longitude 8047′51″ East. It has an area of 972 km2, 21. Ogoja LGA is bordered by Yala LGA to the North and West, Bekwarra LGA to the Northeast, and Obudu LGA and Boki LGA to the East. It is also bordered by Ikom LGA to the South.^20^, ^21^. The region normally has a tropical climate with distinct dry (November to March) and wet (April to October) seasons. Rainfall reaches a mean value of 1200 mm annually. Farming is the major source of livelihood.

### Study design

A cross-sectional study was conducted between January and March 2021. School-age children (5-16 years) from primary and secondary schools were selected using the one-stage cluster sampling method. The primary and secondary schools in Ogoja LGA, were randomly selected from the Cross River State Ministry of Education database. Every subject within the selected schools who consented to the study and met the inclusion criteria, were recruited for the study. After being informed of the study's objective and having their parents or guardians sign consent forms, the students/pupils were only then requested to participate. Faecal and urine samples were obtained from participants who consented to the study. Seven schools were chosen from two communities, Mbube and Nwang, in Ogoja LGA. For ethical reasons, the schools were coded using appropriate codes to avoid providing complete details to the public. The selected schools include Ntol Comprehensive Secondary School, NdokNCSSN; Government Migrant Science and Technology College, MbubeGMSTCM; Christ the King Primary SchoolCKPSM, Mbube; Abi Iruan Primary School, MbubeAIPSM; Community Primary School, EshanjokCPSE; Community Secondary School, NwangCSSN and Saint Theresa Community Primary School, NwangSTCPSN. Baseline data on the prevalence of schistosomiasis were obtained from the Cross River State Neglected Tropical Disease survey conducted in 2014 and 2018. Each respondent's parasitological data were recorded alongside their attendance at school, as determined by the head teacher or principal of each facility. Regular school attendance was defined as being present on at least 60% of instructional days.

### Ethical considerations

Ethical clearance was obtained from the Ministry of Health, Health Research Ethics Committee, Cross River State (CRS/MH/HREC/018/Vol.V1/149). Principals and headmasters/headmistresses of participating schools were informed of the nature of the study, and authorisation to carry out the study in the designated schools was obtained. Additionally, parents and guardians of the participating schoolchildren provided their informed consent.

### Sample size determination

The sample size was estimated using the formula N = Z2 (PQ/I2) described by Bruno and Omar,^22^; where N = sample size, I = 0.05 (margin error), q = 1 − p, z = standard normal deviation set at 1.96 for 5% significance level and p = baseline prevalence of urogenital schistosomiasis in the study area (45%)^13^. A sample size of approximately 380 was determined for the study. However, a total of 504 participants were enrolled in this study to increase the margin of accuracy of the estimates anticipate factors such as voluntary withdrawal or absence during days of sample collection.

### Inclusion and exclusion criteria

This study was designed to target school-age children (5-14 years). The study only included participants who had taken praziquantel at least a year before. Additionally, persons who chose to participate and had resided in the study area for at least a year were included in the study. People who refused to partake in the study, did not provide their permission and did not provide a urine or faecal sample following the interview was were not included in the study. Also, all school-age children that did not fall within the age range of 5-14 were excluded in the study.

**Examination of urine and faecal samples for** *Schistosoma spp*.

Urine samples were collected in properly labeled sterile sample bottles between 10:00 am and 2:00 pm from the participants because egg excretion is highest at approximately midday. All samples were examined using the centrifugation technique. Approximately 10 ml of each urine sample was centrifuged at 2500 rpm for 2 minutes. The supernatant was decanted, and the sediment was examined by light microscopy. A micropipette was used to introduce approximately 10 µl of each sample onto a clean, grease-free glass slide (75 × 25mm) and then covered with a glass slip (22 × 22 mm). A drop of Lugol's solution was added to enhance the identification and counting of eggs. Microscopic examination was carried out using the 10× objective lens magnification. The number of eggs discovered in the preparation was counted and reported as the number of eggs in 10 mL of urine (eggs/10 mL of urine) to represent the intensity of infection^23^. Light (50 eggs/10 mL of urine) or heavy (>50 eggs/10 mL of urine) infections were categorised according to WHO standards^24^. Using a wooden applicator stick, fresh faecal samples for Schistosoma mansoni screening were obtained from each participant and placed in labeled, dry, leak-proof, and sterilized specimen containers. Faecal samples were processed using the Kato-Katz technique (Kato-Katz stool examination kit, Vestergaard Fradson, Switzerland). A 41.7mg template was used^25^. To estimate the intensity in eggs per gram (EPG), the number of eggs was counted and multiplied by a factor of 24. S. mansoni was classified as light (1 – 99 EPG), moderate (100 – 399 EPG), and heavy (≥400 EPG) intensity^24^

### Knowledge Attitude and Practices (KAP)

Data on the Knowledge, Attitude and Practice (KAP) of school-age children concerning schistosomiasis and soil-transmitted helminthiasis were gathered by an interviewer using structured questionnaires (Supplementary data). Parents/guardians of the study participants were present during the interview. The questionnaire covered household socio-demographics, personal hygiene, environmental sanitation practices, and knowledge on soil-transmitted helminthiasis and schistosomiasis. It focused on school-age children in selected schools and included identification numbers for each participant.

### Data analysis

The SPSS package (SPSS, Chicago, IL, USA) version 22 was used for statistical analysis of the data obtained in the study. Calculations of the prevalence and intensity of infections were made using descriptive statistics. The chi-square test was used to determine whether there were any disparities between the variables' data and the outcomes of the sample examinations. To compare the average infection intensity by egg count in the various variables, analysis of variance (ANOVA) was utilised. The strength of the connection between covariables and schistosomiasis was assessed using an odds ratio (OR) with a 95% confidence interval (CI). Graphs were designed using Prism GraphPad (version 8). Differences and associations were considered significant at p≤ 0.05.

## Results

### Prevalence of schistosomiasis among school-age chidren

Five hundred and four (504) faecal and urine samples each were examined each for intestinal and urogenital schistosomiasis respectively. Forty-nine samples [49/504 (9.7%)] were positive for schistosomiasis; 45 (8.9%) and 4 (0.8%) were positive for urogenital and intestinal schistosomiasis respectively (Table 1).

**Table 1:** Prevalence of schistosomiasis among school-age children according to schools surveyed in Ogoja Local Government Area

Schools	Number examined	*S. haematobium* n (%)	*S. mansoni* n (%)	Mixed infection	*p*-value
NCSSN	72	9 (12.5)	1 (1.4)	10 (13.9)	0.261
GMSTCM	70	7 (10.0)	1 (1.4)	8 (11.4)	
CKPSM	69	8 (11.6)	2 (2.9)	10 (14.5)	
AIPSM	74	5 (6.8)	0 (0)	5 (6.8)	
CPSE	66	4 (6.1)	0 (0)	4 (6.1)	
CSSN	70	8 (11.4)	0 (0)	8 (11.4)	
STCPSN	83	4 (4.8)	0 (0)	4 (4.8)	
Total	504	45 (8.9)	4 (0.8)	49 (9.7)	

**Figure 2:**
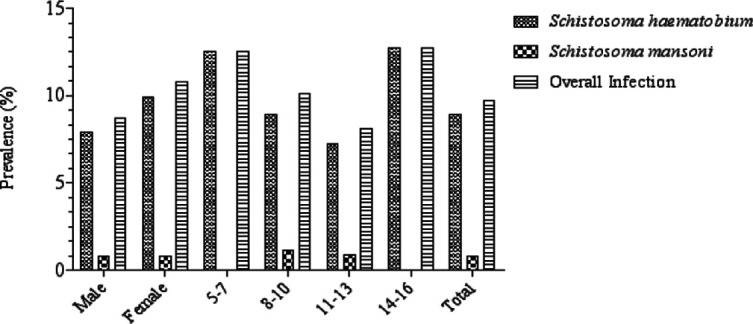
Prevalence of schistosomiasis by gender and age group among school-age children in Ogoja Local Government Area

Infection with Schistosoma haematobium was observed to be highest in Ntol Comprehensive Secondary School-NCSSN (12.5%) and lowest in St. Theresa Community Primary School, NwangSTCPSN (4.8%). S. mansoni infection was low across the different schools and was only present in Ntol Comprehensive Secondary School-NCSSN (1.4%), Government Migrant Science and Technology CollegeGMSTCM (1.4%), and Christ the King Primary School, Ndok (2.9%). School-age children from Christ the King Primary school, NdokCKPSM, recorded the highest overall prevalence of schistosomiasis (14.5%) although this was not significantly different (p=0.261) from the overall prevalence recorded in other schools (Table 1). The overall prevalence of schistosomiasis among males (8.7%) and females (10.8%) showed no significant variation (p=0.435) (Figure 2). Females recorded a higher prevalence of Schistosoma haematobium (9.9%) infection than males (7.9%) (p=0.419). Male and female participants recorded an equal prevalence of S. mansoni infection (0.8%) (Figure 2).

Participants in the age-group 14-16 years showed the highest prevalence of Schistosoma haematobium infection (12.7%) followed closely by participants in the 5-7 years age group (12.5%). The least prevalence was observed in the 11-13 years age group (7.2%) (p=0.462). S. mansoni infection was only recorded in age groups 8-10 and 11-13 years (Figure 2).

### The intensity of schistosomiasis infections by gender and age group among school-age children

Overall, the mean parasite load for urogenital schistosomiasis was 6.40 eggs/10 mL of urine and 36.00 eggs per gram (EPG) for intestinal schistosomiasis. The intensities of Schistosoma mansoni(48.00 EPG) and S. haematobium (7.00 eggs/10 mL) infections were observed to be higher in males than in females (p P>0.05). Between the age groups, the infection intensities of S. mansoni (48.00 EPG) and S. haematobium (8.44eggs/10 mL) were higher among school-age children aged 11-13 years (p P>0.05) (Table 2). All participating school-age children had light infections according to WHO classification.

**Table 2:** Intensity of schistosomiasis in relation to gender and age group among school-age children examined in Ogoja Local Government Area

Category	Number examined	*S. haematobium* (eggs per 10ml urine)	*S. mansoni* (eggs per gram stool)
Gender			
Male	253	7.00 ± 1.518	48.00 ± 24.000
Female	251	5.88 ± 1.395	24.00 ± 0.000
Total	504	6.40 ± 1.019	36.00 ± 12.000
P value		0.588	0.423
Age-group			
5-7	32	4.50 ± 1.323	-
8-10	178	5.47 ± 1.312	24.00 ± 0.000
11-13	223	8.44 ± 2.300	48.00 ± 24.000
14-16	71	5.25 ± 1.849	-
***p*** value		0.744	0.423

### Risk factors associated with schistosomiasis in school-age children

Out of 504 school-age children screened for schistosomiasis infections, the necessary information needed for the study were provided by 355 respondents only, giving a response rate of 70.4%. Others did not give their consent to participate in this part of the study. A high percentage of school-age children in the study had heard about schistosomiasis (69.9%) and the causes of this infection (50.1%). However, infection with schistosomiasis was significantly higher among those who had not heard about schistosomiasis (17.8%) (p=0.000) and who did not know the cause of the infection (12.4%) (p=0.002). About 60% of the participants knew how schistosomiasis is transmitted and this corresponded with a lesser percentage being positive for schistosomiasis (3.8%), compared to participants who did not know about schistosomiasis transmission (14.7%) (p=0.000). Furthermore, participants showed good knowledge of schistosomiasis control given that 67.9% regarded schistosomiasis as a serious disease, 57.2% thought schistosomiasis could be prevented and 67.3% were aware of the source of infection (Table 3).

**Table 3:** Knowledge of schistosomiasis among school-age children surveyed in Ogoja Local Government Area

Variables	No. examined (%) n=355	No. Negative for schistosomiasis (%)	No. Positive for schistosomiasis (%)	*p* value
**Heard about schistosomiasis**				
Yes	248 (69.9)	238 (96.0)	10 (4.0)	
No	107 (30.1)	88 (82.2)	19 (17.8)	0.000
**Causes of schistosomiasis**				
Worms	178 (50.1)	171 (96.1)	7 (3.9)	
Others	177 (49.9)	155 (87.6)	22 (12.4)	0.002
**Signs and symptoms**				
Blood in urine	73 (20.6)	69 (94.5)	4(5.5)	
Do not know	282 (79.4)	257 (91.1)	25 (9.9)	0.134
**How is schistosomiasis transmitted**				
Swimming/bathing in infested water	212 (59.7)	204 (96.2)	8 (3.8)	
Do not know	143 (40.3)	122 (85.3)	21 (14.7)	0.000
**Is schistosomiasis a serious disease**				
Yes	241 (67.9)	237 (98.3)	4 (1.7)	
No	114 (32.1)	89 (78.1)	25 (21.9)	0.000
**Can schistosomiasis be prevented**				
Yes	203 (57.2)	198 (97.5)	5 (2.5)	
No	152 (42.8)	128 (84.2)	24 (15.8)	0.000
**Is faeces/urine a source of infection**				
Yes	239 (67.3)	236 (98.7)	3 (1.3)	
No	116 (32.7)	90 (77.6)	26 (22.4)	0.000

Swimming/bathing in open water (OR = 1.199, 95% CI: 1.027 – 1.400) and fetching water from streams/rivers (OR = 1.202, 95% CI: 1.052 – 1.373) were risk factors significantly associated with schistosomiasis (p P<0.05) (Table 4). Children with schistosomiasis were more likely to have parents/guardians who had no formal education (OR = 2.722, 95% CI: 0.856 – 8.654) (p=0.041), than children whose parents/guardians had postsecondary education. Unemployed parents/guardians (OR = 2.904, 95% CI: 1.064 – 8.126) (p=0.031) had children who were approximately 3.0 times more likely to be infected with schistosomiasis than those who had formal employment. There was no significant association between schistosomiasis infection and school attendance among school-age children in the study (Table 4).

**Table 4:** Risk factors for schistosomiasis infection among school-age children in Ogoja Local Government Area

Variables	No. examined (%) n= 355	No. Negative (%)	No. Positive (%)	OR (CI, 95%)	*p*-value
**Bathe in open water**					
Yes	309 (87.0)	290 (93.9)	19 (6.1)	1.199 (1.027 – 1.400)	0.001
No	46 (13.0)	36 (78.3)	10 (21.7)		
**Fetch water from stream/river**					
Yes	294 (82.8)	278 (94.6)	16 (5.4)	1.202 (1.052 – 1.373)	0.000
No	61 (17.2)	48 (78.7)	13 (21.3)		
**Wash clothes in stream/river**					
Yes	196 (55.2)	178 (90.8)	18 (9.2)	1.025 (0.978 – 1.075)	0.085
No	159 (44.8)	148 (93.1)	11 (6.9)		
**Level of education**					
No formal education	48 (13.5)	34 (70.8)	14 (29.2)	2.722 (0.856 – 8.654)	0.041
Primary education	88 (24.8)	81 (92.0)	7 (7.9)	1.031 (0.894 – 1.189)	0.255
Secondary education	191 (53.8)	186 (97.4)	5 (2.6)	1.091 (0.957 – 1.243)	0.057
Post-secondary education	28 (7.9)	25 (89.3)	3 (10.7)		
**Occupation**					
Farmers	293 (82.5)	276 (94.2)	17 (5.8)	1.069 (0.953 – 1.199)	0.082
Unemployed	20 (5.6)	13 (65.0)	7 (35.0)	2.940 (1.064 – 8.126)	0.031
Formal employment	42 (11.8)	37 (88.1)	5 (11.9)		
**School attendance**					
Regular	176	151 (85.8)	25 (14.2)		
Not regular	31	24 (77.4)	7 (22.6)	1.108 (0.908 – 1.353)	0.099

## Discussion

Data on the demography and hygienic conditions of school-age children in schools are necessary for the development of school health programmes and sustainable control of Neglected Tropical Diseases (including soil-transmitted helminthiases and schistosomiasis)^26^. Although Ogoja LGA has benefitted from the Mass Drug Administration (MDA) of praziquantel for schistosomiasis reports still show continued transmission of the disease in the study area. The findings from this study show that Ogoja LGA in Cross River state is endemic to urogenital and intestinal schistosomiasis. The overall prevalence of urogenital schistosomiasis (8.9%) falls within the stipulated range among LGAs of Cross River State (0 – 32.8%), as previously reported by the Nigerian Federal Ministry of Health^13^. The prevalence of urogenital schistosomiasis (8.9%) was lower than the 13.15% reported in 2014 and the 12.3% in 2018 for Ogoja LGA in a survey conducted by the Cross River State Neglected Tropical Disease centre. The study showed a reduction in the prevalence of urogenital schistosomiasis (8.9%) in comparison to a survey carried out by Adie et al.,^17^ in Ogoja LGA, Cross River State who reported 36% as the prevalence of urogenital schistosomiasis Ogoja LGA. It is likely that the reduction in the prevalence of urogenital schistosomiasis recorded in the study area is a result of the ongoing deworming exercise involving the Mass Drug Administration of praziquantel to school-age children. Prior to this survey, the study area had undergone five rounds of Mass Drug Administration of praziquantel. The benefits of applying mass chemotherapy in the control of schistosomiasis on a large scale in various endemic communities have previously been reported^11^. Similar to the present finding, Adie et al.,^27^ reported a significant reduction in urogenital schistosomiasis infection after the Mass Drug Administration of praziquantel in Biase and Yakurr LGAs in Cross River state. Similar results have also been reported in Sierra Leone^28^ and Burkina Faso^29^.

However, this result is limited sinceonly one stool and urine sample was analysed during the study. This may lead to reduced sensitivity, false negatives, sampling bias, and inadequate monitoring of interventions. A more comprehensive sampling strategy involving multiple samples over time and from diverse locations is recommended to obtain more accurate and reliable results.In situations as reported in this study where the overall prevalence is less than 10%, the WHO recommends case-specific treatment. However, the health care centres in the study area were observed to have inadequate diagnostic tools to carry out proper diagnosis and subsequent treatment. This is of particular importance, as infected individuals left untreated could serve as a source of reinfection in the community.

Schistosoma haematobium infection has been recorded in other LGAs in Cross River state^4^,^5^,^17^,^27^.

Nigeria^6^,^20^,^30^-^32^, and other parts of Africa^7^,^33^,^34^. The overall prevalence of 8.9% recorded for urogenital schistosomiasis is similar to results obtained by Ezeadila et al.,^35^ and Alozie and Anosike^36^ in Enugu and Abia states, respectively. This low prevalence may be linked to the availability of potable water in the study area, although some residents still preferred to obtain water from rivers and streams, thereby risking exposure to schistosomiasis. A higher prevalence of urogenital schistosomiasis infection has been reported by Kabiru et al.,^30^ (38.3%), Tchuem-Tchuenté et al.,^37^ (62.8%), Noriode et al.,^38^ (65.3%) and Adeyemi et al.,^39^ (56%). This high prevalence of infection is linked to the nonavailability of potable water and good sanitary amenities in communities. Individuals in such communities, therefore, depend on water bodies for their water needs. People living in endemic regions run the danger of contracting numerous infections from the continuous dependence on rivers and streams as a source of drinking water. Infection with Schistosoma mansoni was generally low (0.8%), and Oluwole et al.,^40^ stated that the high prevalence of S. haematobium in Nigeria compared to S. mansoni may be due to the ecology and distribution of the snail intermediate hosts, which indirectly affect the distribution of the parasites.

Females recorded a higher prevalence of schistosomiasis (10.8%). This may be a result of sociocultural beliefs, which see female children involved more in fetching water from streams. Such bodies of water may harbour infective cercariae. Similar findings have been reported by Ekpo et al.,^41^ in Abeokuta, Akinboye et al.,^42^ in Ibadan, and Aribodor et al.,^32^ in Enugu. Previous studies, however, have indicated a higher prevalence of schistosomiasis among males^30^,^43^. Kabiru et al.,^30^ reported that males were more infected than females due to more recurrent water contact in cercariae-infested areas and cultural and religious beliefs restricting the role of females solely to housewives and mothers.

Age groups 5-7 years and 14-16 years showed the highest prevalence of Schistosoma haematobium infection (12.5% and 12.7%, respectively). Findings have shown that the prevalence of urogenital schistosomiasis increases as age increases, peaking at age groups>14 years^40^,^44^. This age group (14-16 years) is known to be very active and take part in leisure and agricultural activities in cercariae-infested water which exposes them to S. haematobium and S. mansoni infections. The 5-7 year age group recorded a similar prevalence of S. haematobium infection, which suggests that the contribution of age to the risk of S. haematobium infection may be hinged on other contributing factors, such as parental economic and social status and educational level. A similar result reported by Gbalegba et al.,^45^ stated that children aged 5-7 years were more infected than other age groups because they were not yet targeted by preventive chemotherapy. This is true as most deworming exercises target school children, leaving out those not enrolled in schools. All participating school-age children had light schistosomiasis infection according to the WHO classification^24^. The shows that the elimination of schistosomiasis in Ogoja LGA is on course following the newly released WHO 2021–30 road map for NTD in endemic regions^46^. The low intensity recorded in this study can be attributed to the existence of a potable water supply in the communities. Nevertheless, activities such as farming, dependence on rivers/streams for water needs, and the unemployment status of parents/guardians, have maintained the transmission of the disease in the area. Low-intensity schistosomiasis infection was also reported by Tobin et al.,^47^ in a study conducted in a rural community in South-South Nigeria. However, Noriode et al.,^38^, and Ogbonna et al.,^48^ both reported high intensities of S. haematobium infection in Edo and Enugu states, respectively. This high intensity was caused by the absence of a reliable supply of potable water and adequate sanitary facilities in the study area, which forced people to rely primarily on water courses for their water demand. The intensity of Schistosoma haematobium and S. mansoni infections was observed to be higher in males.

Further studies will be needed to investigate the potential causes of gender disparities in the severity of worm-specific infection among school-age children^49^.

Attitude towards the control of schistosomiasis was poor among school-age children in the study area. Children who reported swimming/bathing in open water and fetching water from the stream were more likely to be infected with schistosomiasis. Although the school-age children had a good knowledge of the transmission of schistosomiasis infection, it did not reflect a change in their behaviour as they still participated in activities that exposed them to the infection.

Open water bathing and collecting water from rivers and streams were strongly linked to schistosomiasis infection. Other research has established the connection between residential water sources and schistosomiasis infection^43^,^50^. According to a survey, rivers, dams, and ponds in endemic regions may serve as possible sites for schistosomiasis infection. Additionally, children with a habit of bathing and washing clothes in rivers and dams had a high rate of schistosomiasis infection. Nevertheless, washing clothes in streams/rivers was not significantly associated with schistosomiasis infection, even though studies have recognised this as a risk factor for schistosomiasis infection^43^,^51^.

Children with schistosomiasis infection were more likely to have parents/guardians who had no formal education than children whose parents/guardians had postsecondary education. This may be explained by the fact that parents with higher education can better comprehend prevention initiatives and communicate this to their children^52^. Parents/guardians who were unemployed had children who were approximately 3 times more likely to be infected with schistosomiasis than those who had formal employment. It is known that individuals who are unemployed mostly resort to farming to meet their basic needs. Participants in the study area engage in rice farming, and rice farming activities have been linked to a high prevalence of schistosomiasis^53^. Houmsou et al.,^54^ reported that children of farmers were more at risk of schistosomiasis infection than children of other occupational groups, perhaps because they assisted their parents in farm work. Furthermore, unemployment has been identified as a marker of low income and poverty, both of which have shown a notable correlation with urogenital schistosomiasis^55^.

Additionally, it has been noted that helminth infection has an impact on school attendance since infected children are more prone to miss school^24^. Children who attended school frequently and those who did not substantially differ in their prevalence of schistosomiasis. On the other hand, research has shown that school-age children who do not consistently attend school have a higher prevalence of schistosomiasis^56^-^58^. According to reports, school attendance declines as helminth infections become more severe, which has a negative impact on the academic achievement of the schoolchildren who are affected^59^. Hence, the positive result obtained in this study can be linked to the general low intensity of schistosomiasis recorded in the study.

## Conclusion

The findings from this study show that Ogoja LGA in Cross River State is still endemic to schistosomiasis after 5 years of the Mass Drug Administration of praziquantel by the National Neglected Tropical Disease Programme- with a higher prevalence recorded in females than males. This means that more sustainable interventions such as the provision of potable water supply, sanitation, and hygiene education, need to be put in place for control to be effective. Even though infection intensities were typically low, the study area still needs urgent integrated control efforts to minimize helminth infection. These measures should focus on health education, providing clean drinking water, vector control, proper disposal of human excreta and sewage, and reducing poverty. Environmental sanitation and hygiene are very important indicators in the control of helminth infection. Deworming programmes should focus on treating out-of-school children and community members at risk as these groups can serve as sources of reinfection.

## Limitations of the study

One faecal and urine sample were obtained from the study participants because of financial constraints since this is not a funded study. Furthermore, coverage information for Mass Drug Administration campaigns in the study area were not provided by the state Neglected Tropical Disease centre as such information was classified as confidential.
